# 5-Chloro-3-ethyl­sulfinyl-2-(4-fluoro­phen­yl)-1-benzofuran

**DOI:** 10.1107/S1600536810001728

**Published:** 2010-01-20

**Authors:** Hong Dae Choi, Pil Ja Seo, Byeng Wha Son, Uk Lee

**Affiliations:** a Department of Chemistry, Dongeui University, San 24 Kaya-dong Busanjin-gu, Busan 614-714, Republic of Korea; b Department of Chemistry, Pukyong National University, 599-1 Daeyeon 3-dong, Nam-gu, Busan 608-737, Republic of Korea

## Abstract

In the title compound, C_16_H_12_ClFO_2_S, the 4-fluoro­phenyl ring is rotated out of the benzofuran plane, as indicated by the dihedral angle of 6.96 (5)°. The crystal structure exhibits a Cl⋯O inter­action with a Cl⋯O distance of 3.163 (1) Å.

## Related literature

For the crystal structures of similar 2-(4-fluoro­phen­yl)-5-halo-3-methyl­sulfinyl-1-benzofuran derivatives, see: Choi *et al.* (2010**a* ▶,*b* ▶,c* ▶). For the biological activity of benzofuran compounds, see: Aslam *et al.* (2006 ▶); Galal *et al.* (2009 ▶); Howlett *et al.* (1999 ▶). For natural products with benzofuran rings, see: Akgul & Anil (2003 ▶); Soekamto *et al.* (2003 ▶). For a review of halogen bonding, see: Politzer *et al.* (2007 ▶).
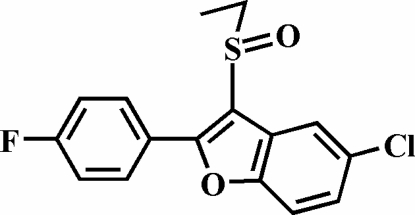


## Experimental

### 

#### Crystal data

C_16_H_12_ClFO_2_S
*M* *_r_* = 322.77
Triclinic, 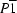

*a* = 7.2843 (1) Å
*b* = 9.4590 (1) Å
*c* = 10.7717 (2) Å
α = 101.630 (1)°
β = 99.301 (1)°
γ = 104.471 (1)°
*V* = 686.08 (2) Å^3^
*Z* = 2
Mo *K*α radiation
μ = 0.44 mm^−1^
*T* = 100 K
0.36 × 0.28 × 0.22 mm

#### Data collection

Bruker SMART APEXII CCD diffractometer
Absorption correction: multi-scan (*SADABS*; Bruker, 2009 ▶) *T*_min_ = 0.685, *T*_max_ = 0.746
10971 measured reflections
2692 independent reflections
2549 reflections with *I* > 2σ(*I*)
*R*_int_ = 0.023

#### Refinement

*R*[*F* ^2^ > 2σ(*F* ^2^)] = 0.033
*wR*(*F* ^2^) = 0.088
*S* = 1.06
2692 reflections
190 parameters
H-atom parameters constrained
Δρ_max_ = 0.53 e Å^−3^
Δρ_min_ = −0.30 e Å^−3^

### 

Data collection: *APEX2* (Bruker, 2009 ▶); cell refinement: *SAINT* (Bruker, 2009 ▶); data reduction: *SAINT*; program(s) used to solve structure: *SHELXS97* (Sheldrick, 2008 ▶); program(s) used to refine structure: *SHELXL97* (Sheldrick, 2008 ▶); molecular graphics: *ORTEP-3* (Farrugia, 1997 ▶) and *DIAMOND* (Brandenburg, 1998 ▶); software used to prepare material for publication: *SHELXL97*.

## Supplementary Material

Crystal structure: contains datablocks global, I. DOI: 10.1107/S1600536810001728/pk2225sup1.cif

Structure factors: contains datablocks I. DOI: 10.1107/S1600536810001728/pk2225Isup2.hkl

Additional supplementary materials:  crystallographic information; 3D view; checkCIF report

## Figures and Tables

**Fig. 1.**
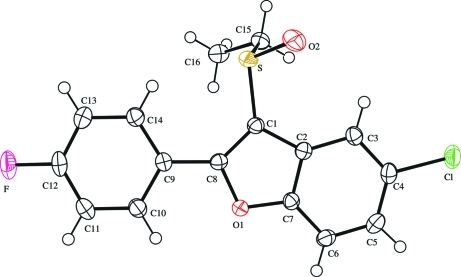
The molecular structure of the title compound with the atom numbering scheme. Displacement ellipsoids are drawn at the 50 % probability level. H atoms are presented as a small spheres of arbitrary radius.

**Fig. 2.**
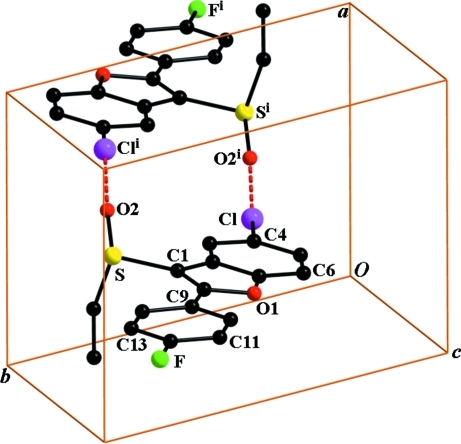
C—Cl···O interactions (dotted lines) in the crystal structure of the title compound. [Symmetry codes: (i) - x + 1, - y + 1, - z.]

## supplementary crystallographic information

### Comment

Molecules involving the benzofuran skeleton have received particular interest in view of their biological activities (Aslam *et al.*, 2006; Galal *et al.*, 2009; Howlett *et al.*, 1999) and their occurrence as natural products (Akgul & Anil, 2003; Soekamto *et al.*, 2003). As a part of our ongoing studies of the effect of side chain substituents on the solid state structures of 2-(4-fluorophenyl)-5-halo-3-methylsulfinyl-1-benzofuran analogues (Choi *et al.*, 2010*a,b,c*), we report the crystal structure of the title compound (Fig. 1).

The benzofuran unit is essentially planar, with a mean deviation of 0.007 (1) Å from the least-squares plane defined by the nine constituent atoms. The dihedral angle formed by the plane of the benzofuran and the 4-fluorophenyl ring is 6.96 (5)°. The crystal packing (Fig. 2) is stabilized by a Cl···O halogen bond between the chlorine and the oxygen of the S═O unit [Cl···O2^i^ = 3.163 (1) Å; C—Cl···O2 = 168.98 (6)°] (Politzer *et al.*, 2007).

### Experimental

77% 3-Chloroperoxybenzoic acid (202 mg, 0.9 mmol) was added in small portions to a stirred solution of 5-chloro-3-ethylsulfanyl-2-(4-fluorophenyl)-1-benzofuran (245 mg, 0.8 mmol) in dichloromethane (30 mL) at 273 K. After being stirred at room temperature for 3h, the mixture was washed with saturated sodium bicarbonate solution and the organic layer was separated, dried over magnesium sulfate, filtered and concentrated in vacuum. The residue was purified by column chromatography (hexane–ethyl acetate, 2:1 v/v) to afford the title compound as a colorless solid [yield 81%, m.p. 413–414 K; *R*_f_ = 0.56 (hexane–ethyl acetate, 2:1 v/v)]. Single crystals suitable for X-ray diffraction were prepared by slow evaporation of a solution of the title compound in acetone at room temperature.

### Refinement

All H atoms were positioned geometrically and refined using a riding model, with C—H = 0.93 Å for aryl, 0.97 Å for methylene, and 0.96 Å for methyl H atoms. *U*_iso_(H) = 1.2*U*_eq_(C) for aryl and methylene H atoms, and 1.5*U*_eq_(C) for methyl H atoms.

### Crystal data

**Table:**

C_16_H_12_ClFO_2_S	*Z* = 2
*M**_r_* = 322.77	*F*(000) = 332
Triclinic, *P*1	*D*_x_ = 1.562 Mg m^−^^3^
Hall symbol: -P 1	Mo *K*α radiation, λ = 0.71073 Å
*a* = 7.2843 (1) Å	Cell parameters from 8228 reflections
*b* = 9.4590 (1) Å	θ = 2.3–27.5°
*c* = 10.7717 (2) Å	µ = 0.44 mm^−^^1^
α = 101.630 (1)°	*T* = 100 K
β = 99.301 (1)°	Block, colourless
γ = 104.471 (1)°	0.36 × 0.28 × 0.22 mm
*V* = 686.08 (2) Å^3^	

### Data collection

**Table:**

Bruker SMART APEXII CCD diffractometer	2692 independent reflections
Radiation source: Rotating Anode	2549 reflections with *I* > 2σ(*I*)
Bruker HELIOS graded multilayer optics	*R*_int_ = 0.023
Detector resolution: 10.0 pixels mm^-1^	θ_max_ = 26.0°, θ_min_ = 2.0°
φ and ω scans	*h* = −8→8
Absorption correction: multi-scan (*SADABS*; Bruker, 2009)	*k* = −11→11
*T*_min_ = 0.685, *T*_max_ = 0.746	*l* = −13→13
10971 measured reflections	

### Refinement

**Table:**

Refinement on *F*^2^	Primary atom site location: structure-invariant direct methods
Least-squares matrix: full	Secondary atom site location: difference Fourier map
*R*[*F*^2^ > 2σ(*F*^2^)] = 0.033	Hydrogen site location: difference Fourier map
*wR*(*F*^2^) = 0.088	H-atom parameters constrained
*S* = 1.06	*w* = 1/[σ^2^(*F*_o_^2^) + (0.0438*P*)^2^ + 0.4635*P*] where *P* = (*F*_o_^2^ + 2*F*_c_^2^)/3
2692 reflections	(Δ/σ)_max_ < 0.001
190 parameters	Δρ_max_ = 0.53 e Å^−^^3^
0 restraints	Δρ_min_ = −0.30 e Å^−^^3^

### Special details

**Table:**

Geometry. All esds (except the esd in the dihedral angle between two l.s. planes) are estimated using the full covariance matrix. The cell esds are taken into account individually in the estimation of esds in distances, angles and torsion angles; correlations between esds in cell parameters are only used when they are defined by crystal symmetry. An approximate (isotropic) treatment of cell esds is used for estimating esds involving l.s. planes.
Refinement. Refinement of F^2^ against ALL reflections. The weighted R-factor wR and goodness of fit S are based on F^2^, conventional R-factors R are based on F, with F set to zero for negative F^2^. The threshold expression of F^2^ > 2sigma(F^2^) is used only for calculating R-factors(gt) etc. and is not relevant to the choice of reflections for refinement. R-factors based on F^2^ are statistically about twice as large as those based on F, and R- factors based on ALL data will be even larger.

### Fractional atomic coordinates and isotropic or equivalent isotropic displacement parameters (Å^2^)

**Table:**

	*x*	*y*	*z*	*U*_iso_*/*U*_eq_	
S	0.45255 (6)	0.80712 (5)	0.40708 (4)	0.02482 (13)	
Cl	0.28282 (7)	0.27078 (5)	−0.05264 (4)	0.03514 (14)	
F	0.25946 (19)	0.84365 (15)	1.02077 (10)	0.0444 (3)	
O1	0.20936 (16)	0.41731 (12)	0.48378 (11)	0.0220 (2)	
O2	0.5927 (2)	0.79848 (16)	0.32129 (14)	0.0365 (3)	
C1	0.3394 (2)	0.62050 (18)	0.41357 (15)	0.0206 (3)	
C2	0.2984 (2)	0.48819 (18)	0.30777 (15)	0.0208 (3)	
C3	0.3201 (2)	0.46061 (19)	0.17918 (16)	0.0237 (3)	
H3	0.3723	0.5387	0.1430	0.028*	
C4	0.2602 (2)	0.3116 (2)	0.10851 (16)	0.0258 (4)	
C5	0.1846 (3)	0.1912 (2)	0.16042 (17)	0.0285 (4)	
H5	0.1486	0.0927	0.1093	0.034*	
C6	0.1631 (3)	0.21813 (19)	0.28749 (17)	0.0258 (4)	
H6	0.1129	0.1399	0.3240	0.031*	
C7	0.2202 (2)	0.36738 (18)	0.35738 (15)	0.0212 (3)	
C8	0.2840 (2)	0.57255 (18)	0.51702 (16)	0.0206 (3)	
C9	0.2817 (2)	0.64559 (19)	0.64935 (15)	0.0213 (3)	
C10	0.2304 (2)	0.5573 (2)	0.73545 (17)	0.0261 (4)	
H10	0.2019	0.4529	0.7085	0.031*	
C11	0.2220 (3)	0.6242 (2)	0.86001 (17)	0.0304 (4)	
H11	0.1849	0.5657	0.9166	0.036*	
C12	0.2694 (3)	0.7788 (2)	0.89875 (16)	0.0304 (4)	
C13	0.3240 (3)	0.8701 (2)	0.81899 (17)	0.0306 (4)	
H13	0.3575	0.9745	0.8485	0.037*	
C14	0.3279 (2)	0.8025 (2)	0.69353 (17)	0.0262 (4)	
H14	0.3620	0.8624	0.6374	0.031*	
C15	0.2417 (3)	0.8367 (2)	0.31295 (17)	0.0275 (4)	
H15A	0.2831	0.9250	0.2801	0.033*	
H15B	0.1812	0.7504	0.2390	0.033*	
C16	0.0937 (3)	0.8588 (2)	0.39391 (19)	0.0300 (4)	
H16A	0.1526	0.9452	0.4664	0.036*	
H16B	0.0505	0.7708	0.4251	0.036*	
H16C	−0.0155	0.8743	0.3413	0.036*	

### Atomic displacement parameters (Å^2^)

**Table:**

	*U*^11^	*U*^22^	*U*^33^	*U*^12^	*U*^13^	*U*^23^
S	0.0245 (2)	0.0222 (2)	0.0283 (2)	0.00369 (16)	0.00953 (17)	0.00866 (16)
Cl	0.0472 (3)	0.0381 (3)	0.0213 (2)	0.0177 (2)	0.00961 (18)	0.00222 (18)
F	0.0576 (8)	0.0547 (8)	0.0209 (5)	0.0212 (6)	0.0129 (5)	0.0003 (5)
O1	0.0256 (6)	0.0205 (6)	0.0219 (6)	0.0071 (5)	0.0080 (4)	0.0070 (4)
O2	0.0333 (7)	0.0367 (7)	0.0437 (8)	0.0072 (6)	0.0211 (6)	0.0138 (6)
C1	0.0200 (8)	0.0200 (8)	0.0226 (8)	0.0061 (6)	0.0062 (6)	0.0059 (6)
C2	0.0187 (7)	0.0223 (8)	0.0226 (8)	0.0081 (6)	0.0050 (6)	0.0052 (6)
C3	0.0234 (8)	0.0275 (9)	0.0226 (8)	0.0098 (7)	0.0072 (6)	0.0071 (7)
C4	0.0265 (8)	0.0315 (9)	0.0214 (8)	0.0136 (7)	0.0063 (6)	0.0041 (7)
C5	0.0303 (9)	0.0240 (8)	0.0296 (9)	0.0114 (7)	0.0039 (7)	0.0012 (7)
C6	0.0274 (8)	0.0210 (8)	0.0303 (9)	0.0083 (7)	0.0068 (7)	0.0072 (7)
C7	0.0213 (8)	0.0234 (8)	0.0215 (8)	0.0095 (6)	0.0063 (6)	0.0062 (6)
C8	0.0187 (7)	0.0200 (8)	0.0241 (8)	0.0068 (6)	0.0051 (6)	0.0060 (6)
C9	0.0184 (7)	0.0258 (8)	0.0203 (8)	0.0075 (6)	0.0043 (6)	0.0057 (6)
C10	0.0275 (9)	0.0284 (9)	0.0242 (8)	0.0093 (7)	0.0067 (7)	0.0082 (7)
C11	0.0324 (9)	0.0401 (10)	0.0237 (9)	0.0131 (8)	0.0095 (7)	0.0135 (8)
C12	0.0298 (9)	0.0432 (11)	0.0183 (8)	0.0152 (8)	0.0057 (7)	0.0019 (7)
C13	0.0320 (9)	0.0289 (9)	0.0280 (9)	0.0096 (7)	0.0065 (7)	0.0005 (7)
C14	0.0271 (9)	0.0269 (9)	0.0248 (8)	0.0070 (7)	0.0077 (7)	0.0063 (7)
C15	0.0343 (9)	0.0255 (9)	0.0254 (8)	0.0103 (7)	0.0072 (7)	0.0100 (7)
C16	0.0256 (9)	0.0284 (9)	0.0375 (10)	0.0088 (7)	0.0076 (7)	0.0102 (7)

### Geometric parameters (Å, °)

**Table:**

Cl—O2^i^	3.163 (1)	C6—H6	0.9300
S—O2	1.489 (1)	C8—C9	1.458 (2)
S—C1	1.772 (2)	C9—C14	1.398 (2)
S—C15	1.8142 (18)	C9—C10	1.402 (2)
Cl—C4	1.745 (2)	C10—C11	1.381 (2)
F—C12	1.355 (2)	C10—H10	0.9300
O1—C7	1.370 (2)	C11—C12	1.373 (3)
O1—C8	1.381 (2)	C11—H11	0.9300
C1—C8	1.367 (2)	C12—C13	1.371 (3)
C1—C2	1.443 (2)	C13—C14	1.382 (2)
C2—C7	1.391 (2)	C13—H13	0.9300
C2—C3	1.399 (2)	C14—H14	0.9300
C3—C4	1.382 (2)	C15—C16	1.520 (2)
C3—H3	0.9300	C15—H15A	0.9700
C4—C5	1.398 (3)	C15—H15B	0.9700
C5—C6	1.383 (2)	C16—H16A	0.9600
C5—H5	0.9300	C16—H16B	0.9600
C6—C7	1.382 (2)	C16—H16C	0.9600
			
C4—Cl—O2^i^	168.98 (6)	C14—C9—C8	121.88 (15)
O2—S—C1	107.41 (8)	C10—C9—C8	119.66 (15)
O2—S—C15	106.84 (8)	C11—C10—C9	120.48 (17)
C1—S—C15	97.19 (8)	C11—C10—H10	119.8
C7—O1—C8	106.92 (12)	C9—C10—H10	119.8
C8—C1—C2	107.26 (14)	C12—C11—C10	118.76 (16)
C8—C1—S	128.13 (13)	C12—C11—H11	120.6
C2—C1—S	124.54 (12)	C10—C11—H11	120.6
C7—C2—C3	119.43 (15)	F—C12—C13	118.64 (17)
C7—C2—C1	105.04 (14)	F—C12—C11	118.45 (17)
C3—C2—C1	135.52 (16)	C13—C12—C11	122.91 (16)
C4—C3—C2	116.69 (15)	C12—C13—C14	118.13 (17)
C4—C3—H3	121.7	C12—C13—H13	120.9
C2—C3—H3	121.7	C14—C13—H13	120.9
C3—C4—C5	123.17 (16)	C13—C14—C9	121.24 (16)
C3—C4—Cl	118.61 (14)	C13—C14—H14	119.4
C5—C4—Cl	118.21 (13)	C9—C14—H14	119.4
C6—C5—C4	120.24 (16)	C16—C15—S	111.69 (12)
C6—C5—H5	119.9	C16—C15—H15A	109.3
C4—C5—H5	119.9	S—C15—H15A	109.3
C7—C6—C5	116.50 (16)	C16—C15—H15B	109.3
C7—C6—H6	121.7	S—C15—H15B	109.3
C5—C6—H6	121.7	H15A—C15—H15B	107.9
O1—C7—C6	125.40 (15)	C15—C16—H16A	109.5
O1—C7—C2	110.66 (14)	C15—C16—H16B	109.5
C6—C7—C2	123.94 (15)	H16A—C16—H16B	109.5
C1—C8—O1	110.11 (14)	C15—C16—H16C	109.5
C1—C8—C9	135.48 (15)	H16A—C16—H16C	109.5
O1—C8—C9	114.40 (13)	H16B—C16—H16C	109.5
C14—C9—C10	118.46 (15)		
			
O2—S—C1—C8	143.68 (15)	C2—C1—C8—O1	−0.18 (18)
C15—S—C1—C8	−106.11 (16)	S—C1—C8—O1	−177.16 (11)
O2—S—C1—C2	−32.81 (16)	C2—C1—C8—C9	−178.51 (17)
C15—S—C1—C2	77.40 (15)	S—C1—C8—C9	4.5 (3)
C8—C1—C2—C7	−0.29 (17)	C7—O1—C8—C1	0.59 (17)
S—C1—C2—C7	176.82 (12)	C7—O1—C8—C9	179.30 (13)
C8—C1—C2—C3	179.92 (18)	C1—C8—C9—C14	6.7 (3)
S—C1—C2—C3	−3.0 (3)	O1—C8—C9—C14	−171.53 (14)
C7—C2—C3—C4	0.2 (2)	C1—C8—C9—C10	−173.73 (18)
C1—C2—C3—C4	179.97 (17)	O1—C8—C9—C10	8.0 (2)
C2—C3—C4—C5	−1.2 (2)	C14—C9—C10—C11	1.4 (2)
C2—C3—C4—Cl	179.67 (12)	C8—C9—C10—C11	−178.16 (15)
C3—C4—C5—C6	1.2 (3)	C9—C10—C11—C12	−1.6 (3)
Cl—C4—C5—C6	−179.67 (13)	C10—C11—C12—F	179.52 (16)
C4—C5—C6—C7	−0.1 (3)	C10—C11—C12—C13	0.4 (3)
C8—O1—C7—C6	178.85 (15)	F—C12—C13—C14	−178.07 (16)
C8—O1—C7—C2	−0.78 (17)	C11—C12—C13—C14	1.1 (3)
C5—C6—C7—O1	179.54 (15)	C12—C13—C14—C9	−1.3 (3)
C5—C6—C7—C2	−0.9 (3)	C10—C9—C14—C13	0.1 (3)
C3—C2—C7—O1	−179.50 (13)	C8—C9—C14—C13	179.63 (16)
C1—C2—C7—O1	0.67 (17)	O2—S—C15—C16	−175.89 (12)
C3—C2—C7—C6	0.9 (2)	C1—S—C15—C16	73.42 (13)
C1—C2—C7—C6	−178.98 (15)		

Symmetry codes: (i) −*x*+1, −*y*+1, −*z*.
